# Supplementary material for: Tumor-intrinsic expression of the autophagy gene Atg16l1 suppresses anti-tumor immunity in colorectal cancer
Source: Nat Commun. 2023 Sep 23;14:5945. doi: 10.1038/s41467-023-41618-7 (PMC10517947; doi:10.1038/s41467-023-41618-7)
Supplement: Supplementary file 1 — Supplementary Information [file 41467_2023_41618_MOESM1_ESM.pdf]

Supplementary Information

Tumor-intrinsic expression of the autophagy gene Atg16l1 suppresses anti-tumor immunity in colorectal cancer

Lucia Taraborrelli[1,#], Yasin Şenbabaoğlu[2,#], Lifen Wang[1,#], Junghyun Lim[1], Kerrigan Blake[1], Noelyn Kljavin[3], Sarah Gierke[4,5], Alexis Scherl[5], James Ziai[5], Erin McNamara[6], Mark Owyong[6], Shilpa Rao[2], Aslihan Karabacak Calviello[2], Daniel Oreper[2], Suchit Jhunjhunwala[2], Guillem Argiles[7], Johanna Bendell[8], Tae Won Kim[9], Fortunato Ciardiello[10], Matthew J Wongchenko[11], Frederic J de Sauvage[3], Felipe de Sousa e Melo[12], Yibing Yan[11], Nathaniel R West[1,*], Aditya Murthy[1,13,*]

Affiliations

[1] Department of Cancer Immunology, Genentech Inc., South San Francisco, United States.
[2] Department of Oncology Bioinformatics, Genentech Inc., South San Francisco, United States.
[3] Department of Molecular Oncology, Genentech Inc., South San Francisco, United States.
[4] Center for Advanced Light Microscopy, Genentech Inc., South San Francisco, United States.
[5] Department of Pathology, Genentech Inc., South San Francisco, United States.
[6] Department of In Vivo Pharmacology, Genentech Inc., South San Francisco, United States.
[7] Vall d'Hebrón Institute of Oncology, Vall d'Hebrón University Hospital, Universitat Autònoma de Barcelona, Barcelona, Spain.
[8] Sarah Cannon Research Institute/Tennessee Oncology, Nashville, TN, United States.
[9] Department of Oncology, Medical Center, University of Ulsan, Seoul, Korea.
[10] Department of Precision Medicine, Università degli Studi della Campania Luigi Vanvitelli, Naples, Italy.
[11] Oncology Biomarker Development, Genentech, Inc., South San Francisco, CA, United States.
[12] Department of Discovery Oncology, Genentech Inc., South San Francisco, United States.
[13] Present address: Gilead Sciences, Foster City, United States.

[#] These authors contributed equally

* These authors jointly supervised this work

Correspondence: N.R.W. west.nathaniel@gene.com, A.M. aditya.murthy@gilead.com

Supplementary Figure 1

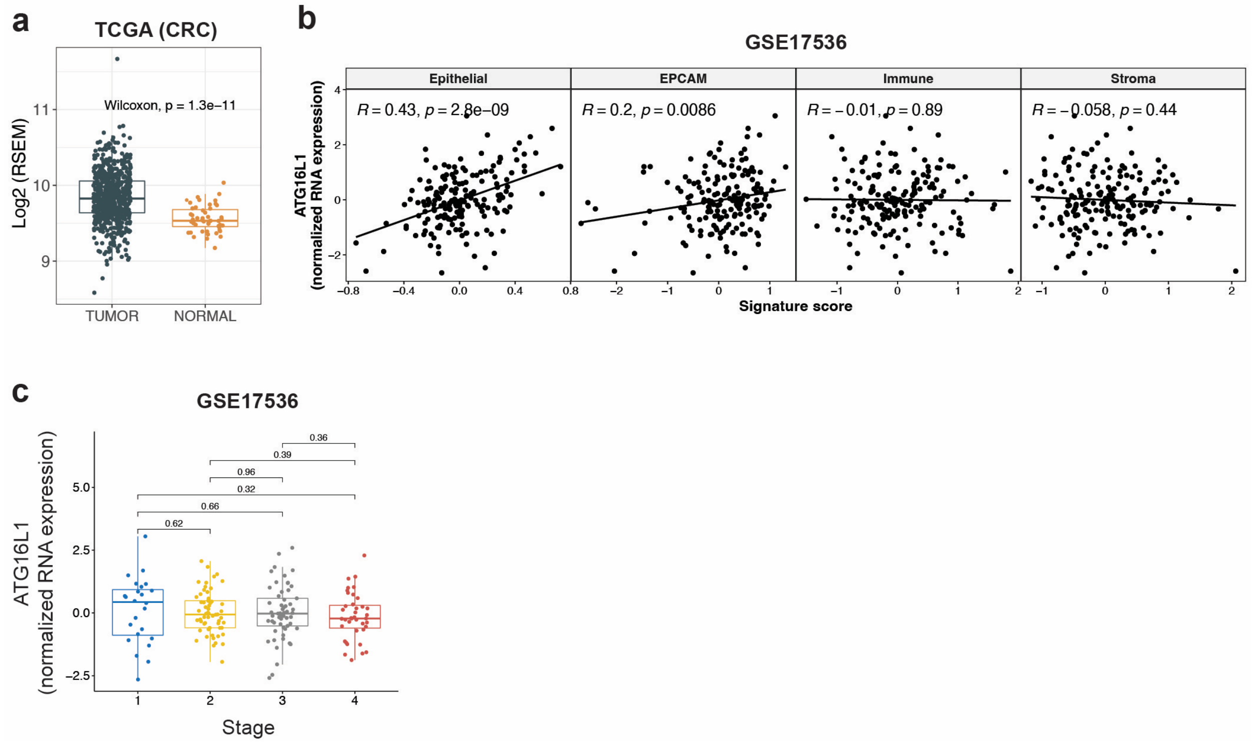

Supplementary Fig.1: *ATG16L1* expression is associated with aggressive disease in human CRC.

a, *ATG16L1* transcript levels in human colorectal cancer (*n*=615 samples) and adjacent normal tissue (*n*=51 samples) from TCGA. Lower and upper hinges in box plots correspond to first and third quartiles, while whiskers extend to 1.5 times interquartile range. **b**, Scatter plots depicting the correlation between *ATG16L1* transcript levels and indicated signatures such as immune, stroma and malignant cells (i.e., epithelial) in samples analyzed from GSE17536. Pearson correlation coefficients and two-sided t-test *P*-values shown (*n*=176 samples). **c**, ATG16L1 transcript levels across stages. Wilcoxon rank-sum test *P*-values shown (*n*=24, 57, 57, and 39 samples for stage 1, 2, 3, 4 patients, respectively). Lower and upper hinges in box plots correspond to first and third quartiles, while whiskers extend to 1.5 times interquartile range.

Supplementary Figure 2

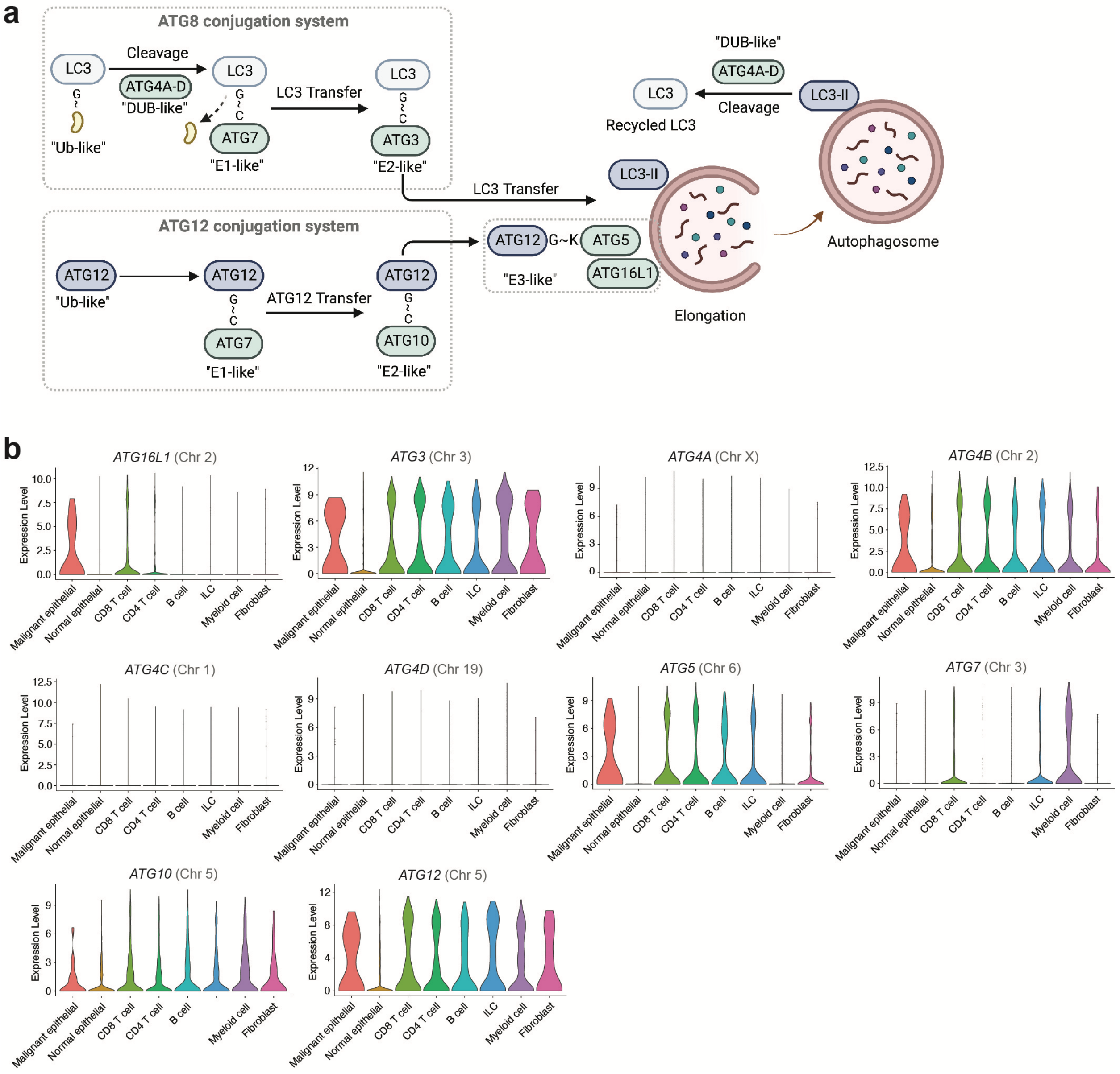

Supplementary Fig. 2: Comparison of gene expression of core autophagosome elongation complex genes in the CRC tumor microenvironment.

a, Illustration of the autophagosome elongation machinery and core components (reviewed in Refs. 1, 2). **b**, Violin plots comparing relative gene expression of indicated *ATG* genes across cellular compartments of the CRC tumor microenvironment. Data derived from GEO dataset GSE146771.

Supplementary Figure 3

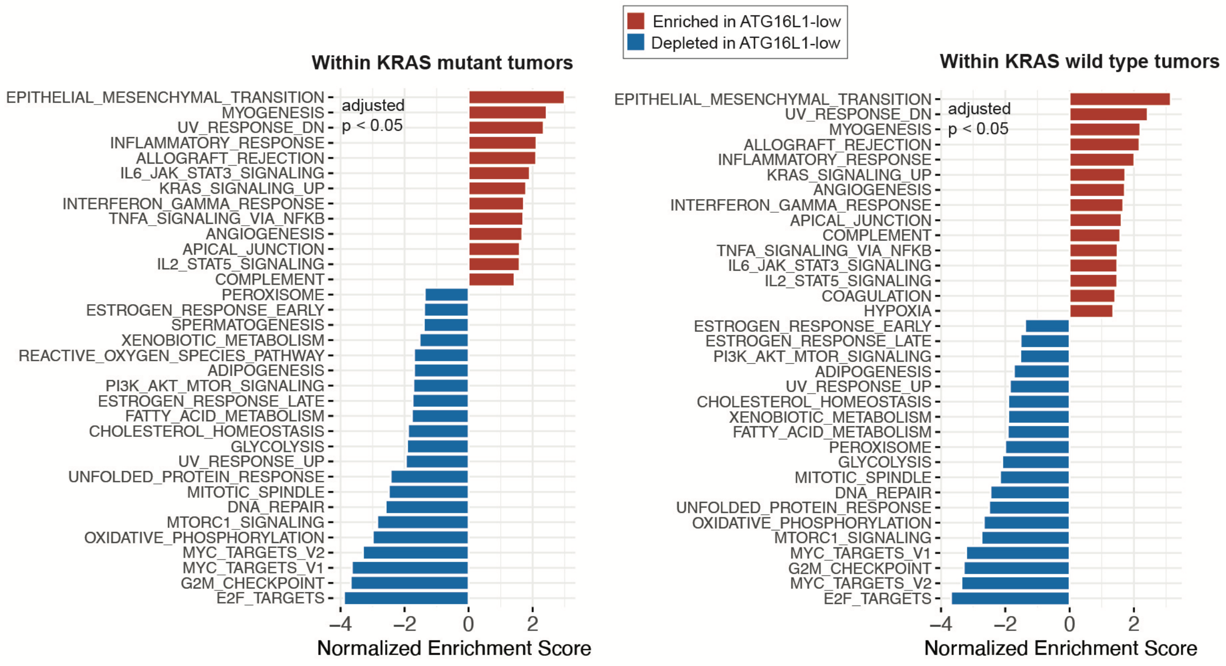

Supplementary Fig. 3: Identification of gene-expression profiles associated with *ATG16L1* transcript levels in KRAS mutant and wild type tumors from IMblaze370. MSigDB hallmark gene set enrichment analysis performed with fgsea. The y-axis shows gene sets significant at false discovery rate-adjusted $P<0.05$. (KRAS mutant $n=181$, KRAS wildtype $n=113$ samples). All analysis restricted to non-MSI-high tumors. $^{*}P < 0.05$, $^{**}P < 0.01$, and $^{***}P < 0.001$.

Supplementary Figure 4

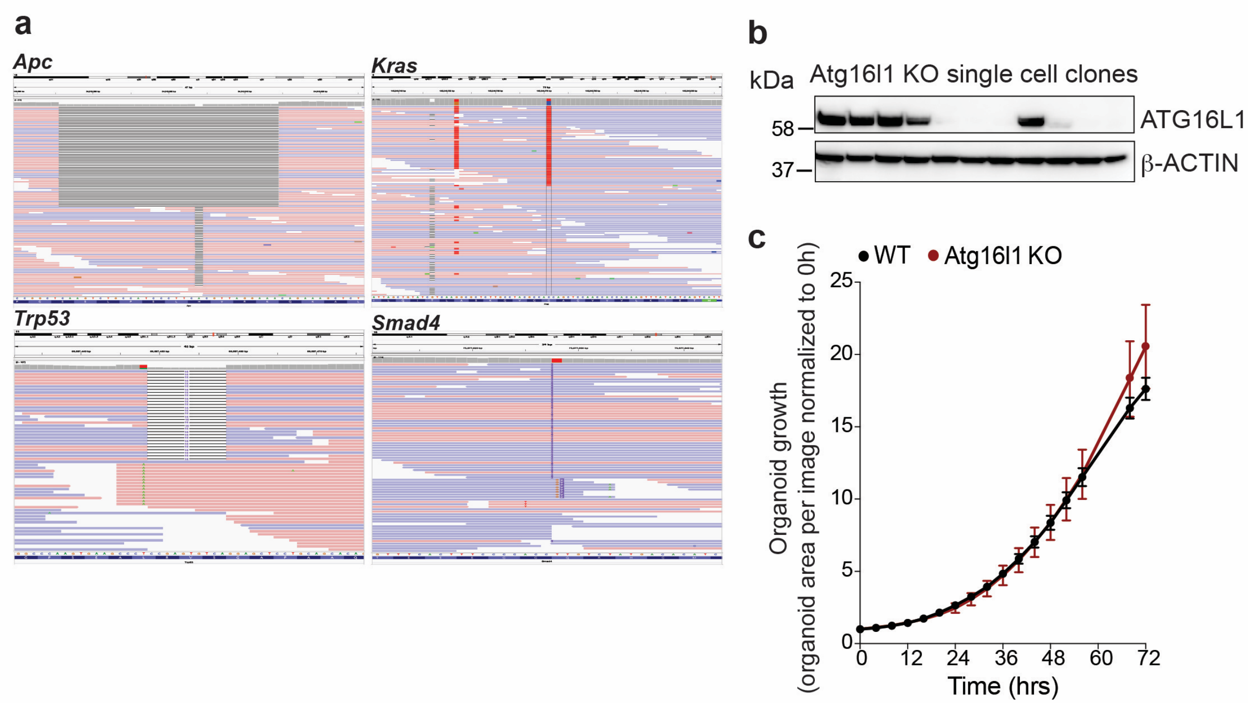

Supplementary Fig. 4: Generation and characterization of Atg16l1 KO CRC organoids.

a, IGV snapshots confirming mutations in the AKPS CRC organoids. Trp53 and Smad4 are knocked out via partial deletion and an insertion, respectively. Apc is knocked out via partial deletion. A heterozygous G12D mutation is introduced into Kras. **b**, Immunoblot analysis of the indicated total proteins in WT or Atg16l1 KO CRC organoids. **c**, Growth of WT or Atg16l1 KO CRC organoids measured via Incucyte for 72 hours. Data are representative of two independent experiments. Source data for panels **b** and **c** are provided as a Source Data file.

Supplementary Figure 5

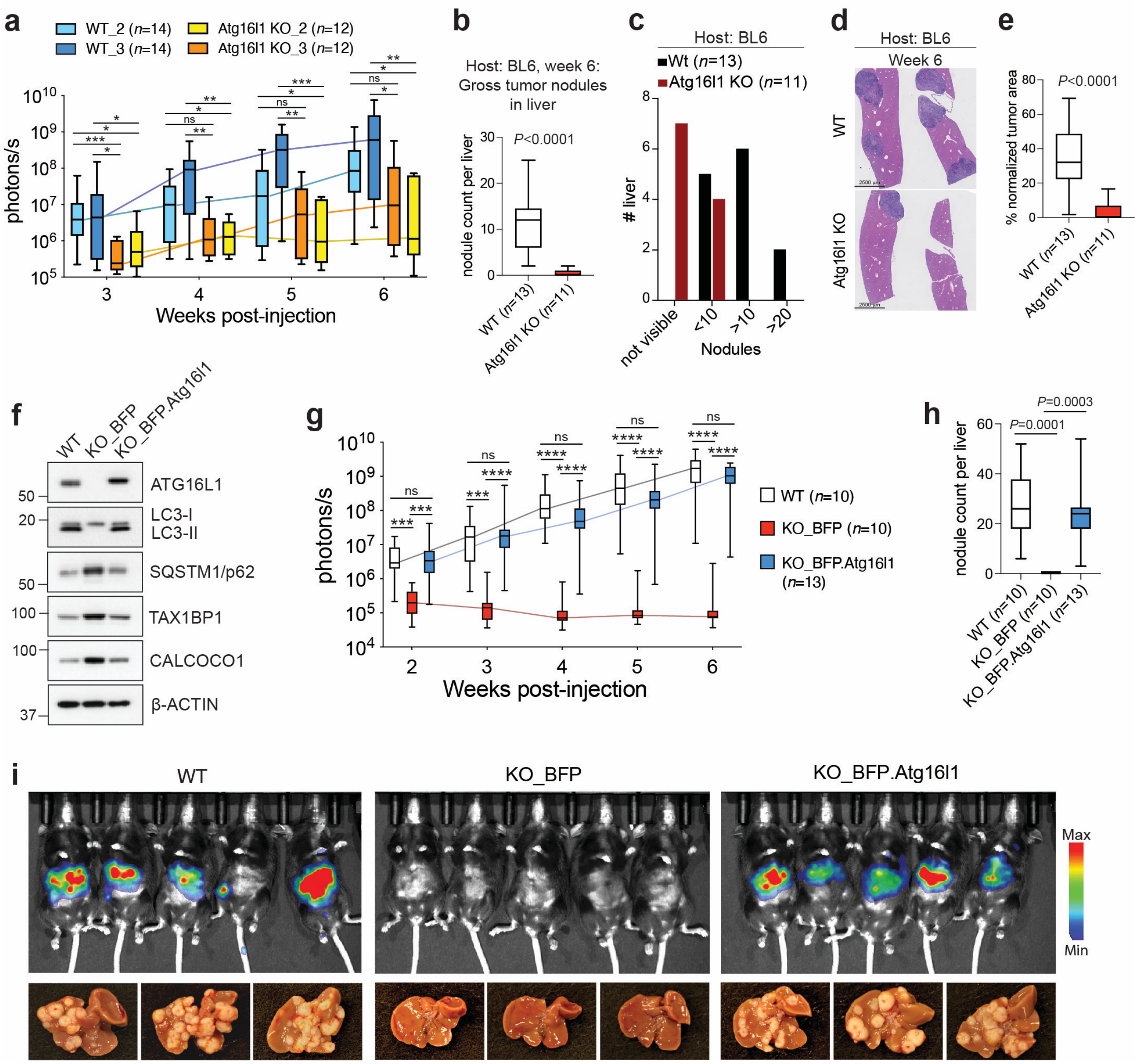

Supplementary Fig. 5: Loss of Atg16l1 in CRC organoids inhibits liver colonization in immunocompetent mice.

a, BLI quantification of independent studies with multiple clones of WT and Atg16l1 KO (KO) CRC organoids pooled into a single growth curve for direct comparison. **b**, **c**, Macroscopic quantification of tumor nodules in the liver of BL6 mice administered with WT or Atg16l1 KO CRC organoids. **d**, Representative images of liver sections stained with H&E of mice administered with WT or Atg16l1 KO CRC organoids. **e**, Quantification of tumor area normalized per total tissue from H&E staining. **f**, Representative immunoblots depicting rescue of ATG16L1 protein expression and autophagic flux markers upon re-expression of ATG16L1 into KO CRC organoids (named

KO_BFP.Atg16l1). Data represent one clone from each condition (corresponding to groups in panels **g**–**i**). **g**, **h**, Tumor BLI quantification (**g**) and macroscopic quantification of tumor nodules (**h**) demonstrating that re-expression of ATG16L1 rescues tumor growth of Atg16l1 KO CRC organoids in immunocompetent BL6 hosts. **i**, Representative BLI images of mice (top) and tumor burden in livers (bottom) from tumors of indicated genotypes grown in immunocompetent BL6 hosts (WT, n=10; KO_BFP, n=10; KO_BFP.Atg16l1, n=13). Box-and-whisker plots depict medians, upper and lower quartiles, maxima, and minima. P-values determined by Mann-Whitney tests with Holm-Sidak multiple testing correction as appropriate (panels **a**, **b**, **e** and **g**) or by Kruskal-Wallis test with Dunnet's multiple comparisons test (**h**). In panels **a** and **g**, *P<0.05, **P<0.01, ***P < 0.001, and ****P < 0.0001. ns, not significant. Source data (including exact P-values for panels **a** and **g**) are provided as a Source Data file.

Supplementary Figure 6

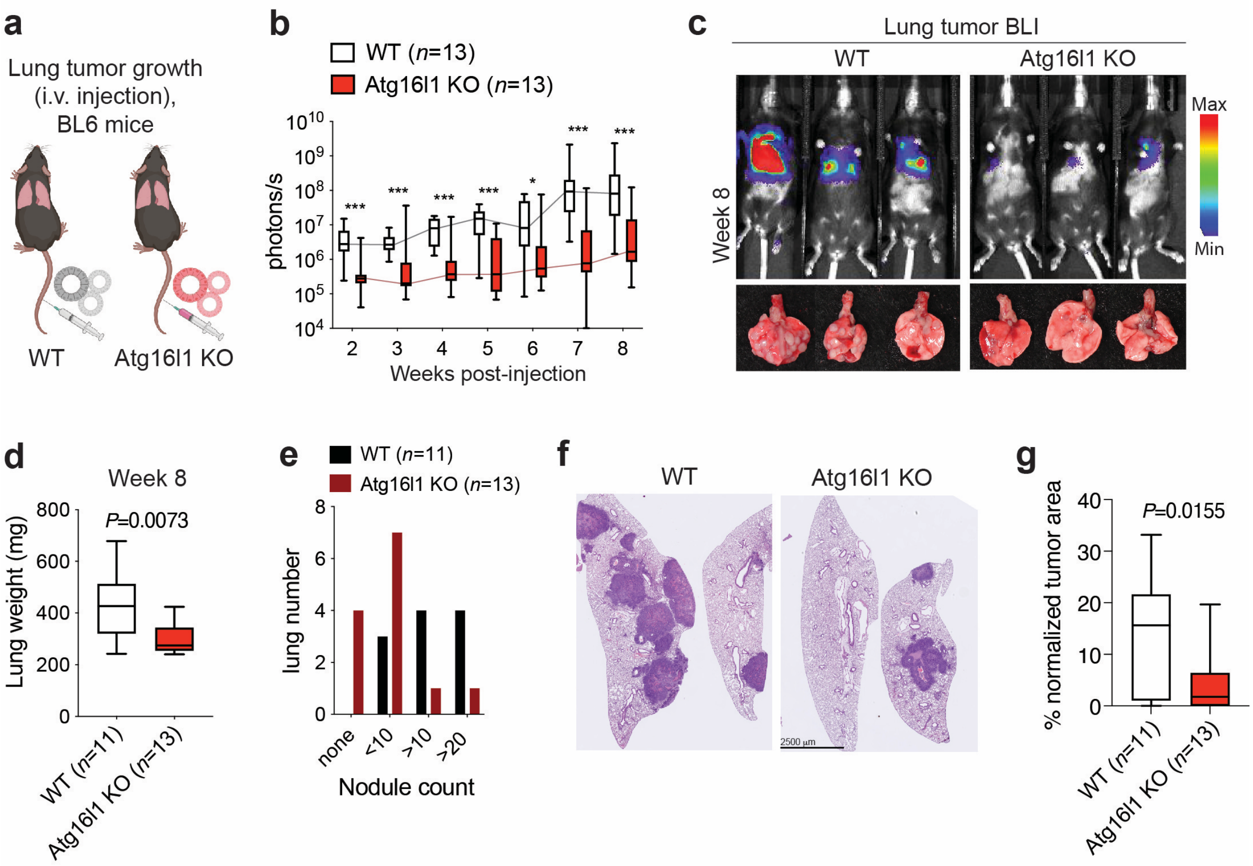

Supplementary Fig. 6: Loss of Atg16l1 in CRC organoids reduces their growth in the lung.

a, Schematic of intravenous (i.v.) tail-vein injection for lung colonization by CRC organoids. **b**, Quantification of thoracic BLI signal of BL6 mice administered with WT or Atg16l1 KO CRC organoids via i.v. injection. **c**, Representative BLI images of mice (top) and lungs (bottom) from BL6 mice administered with WT or Atg16l1 KO CRC organoids via i.v. injection (*n*=13 per group). **d**, Lung weight of BL6 mice administered WT or Atg16l1 KO CRC organoids via i.v. injection. **e**, Macroscopic quantification of tumor nodules in the lungs of mice administered with WT or Atg16l1 KO CRC organoids via i.v. injection. **f**, Representative images of lung sections stained with H&E of mice administered with WT or Atg16l1 KO CRC organoids via i.v. injection. **g**, Quantification of tumor area normalized per total tissue from H&E staining. Data are representative of two independent experiments. Box-and-whisker plots depict medians, upper and lower quartiles, maxima, and minima. *P*-values determined by Mann-Whitney tests with Holm-Sidak multiple-testing correction (**b**) or standard Mann-Whitney tests (**d**, **g**). $^{*}P < 0.05$ and $^{***}P < 0.001$. Source data (including exact *P*-values for panel **b**) are provided as a Source Data file.

Supplementary Figure 7

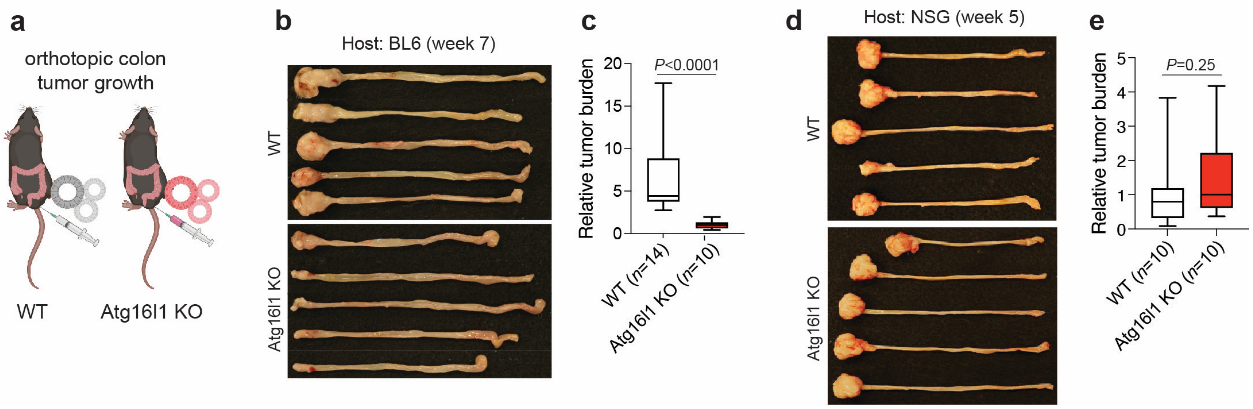

Supplementary Fig. 7: Loss of Atg16l1 in CRC organoids attenuates tumor growth in the colon of immunocompetent mice.

a, Schematic of orthotopic CRC organoid implantation. **b**, **d**, Representative macroscopic images of colon tumors arising from WT or Atg16l1 KO CRC organoids implanted in immunocompetent BL6 hosts at week 7 post-implantation (**b**) or immunocompromised NSG hosts at week 5 post-implantation (**d**). **c**, **e**, Quantification of tumor burden in immunocompetent BL6 **(c)** or immunodeficient NSG (**e**) hosts following orthotopic implantation of WT or Atg16l1 KO CRC organoids. Tumor volumes are normalized to the medians of Atg16l1 KO groups. Box-and-whisker plots depict medians, upper and lower quartiles, maxima, and minima. *P*-values determined by Mann-Whitney test. Source data for panels **c** and **e** are provided as a Source Data file.

Supplementary Figure 8

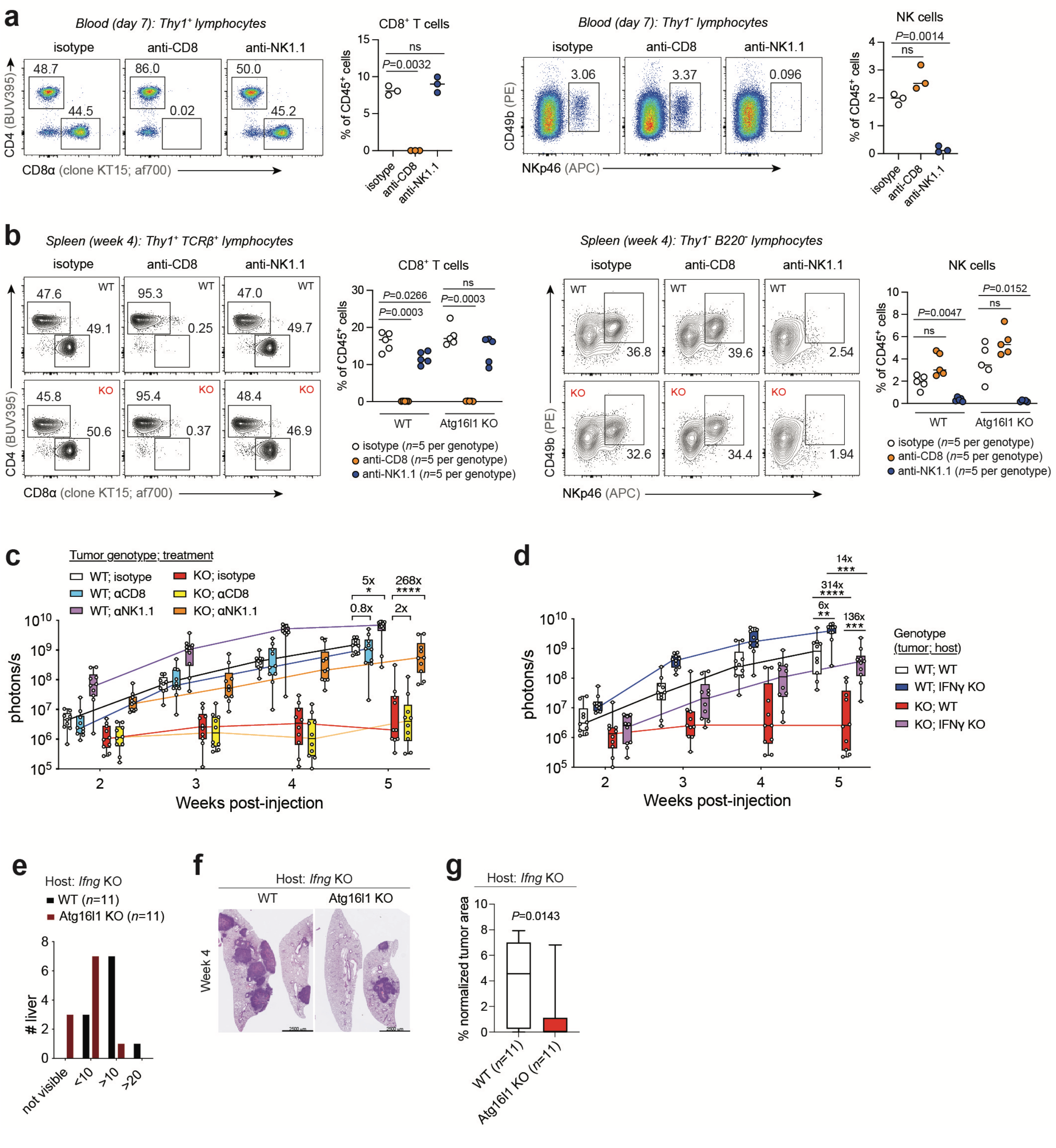

Supplementary Fig. 8: Contribution of adaptive immunity to decreased liver colonization by Atg16l1 KO CRC organoids.

a, Flow cytometry analysis of the frequency of CD8$^+$ T and NK cells in blood of WT BL6 mice 7 days after depletion of indicated immune cell populations. Mice were treated with anti-CD8 or anti-NK1.1 depleting antibodies or with isotype controls. Left, representative plots. Right, quantification of cell frequency (*n*=3 mice per condition). Adjusted *P*-values

are shown, derived from ANOVA with Dunnett's T3 multiple comparisons test. **b**, Flow cytometry analysis of $CD8^+$ T and NK cell depletion in spleen of WT BL6 mice 4 weeks after HTV administration of WT or ATG16L1 KO CRC organoids. Mice were treated with anti-CD8 or anti-NK1.1 depleting antibodies or with isotype controls starting 1 day prior to tumor cell injection. Left, representative plots. Right, quantification of cell frequency (*n*=5 mice per tumor genotype and condition). Adjusted *P*-values are shown, derived from ANOVA with Dunnett's T3 multiple comparisons test. **c**, Quantification of liver BLI signal over 5 weeks in BL6 mice administered WT or Atg16l1 KO CRC organoids via HTV, treated with indicated depletion or isotype control non-depleting antibodies. For all Atg16l1 KO groups, *n*=10 per condition. For WT groups, *n*=9 for isotype control-treated mice, and *n*=10 each for anti-CD8 and anti-NK1.1 treated mice. *P*-values determined by two-tailed Mann-Whitney test. *P=0.0279, ****P<0.0001. Tumor growth of isotype versus anti-CD8-treated mice was not significantly different (P>0.05). **d**, Quantification of liver BLI signal over 5 weeks in BL6 WT or *Ifng* KO mice administered WT or Atg16l1 KO CRC organoids via HTV (*n*=10 mice per group). *P*-values determined by two-tailed Mann-Whitney test. **P<0.01, ***P<0.001, ****P<0.0001. **e**, Macroscopic quantification of tumor nodules in the liver of *Ifng* KO mice administered with WT or Atg16l1 KO CRC organoids. **f**, Representative images of liver sections stained with H&E of *Ifng* KO mice administered with WT or Atg16l1 KO CRC organoids. **g**, Quantification of tumor area normalized per total tissue from H&E staining. *P*-values determined by two-tailed Mann-Whitney test. In all panels, box-and-whisker plots depict medians, upper and lower quartiles, maxima, and minima. All data are representative of 2–3 independent studies. Source data (including exact *P*-values for panel **d**) provided as a Source Data file.

Supplementary Figure 9

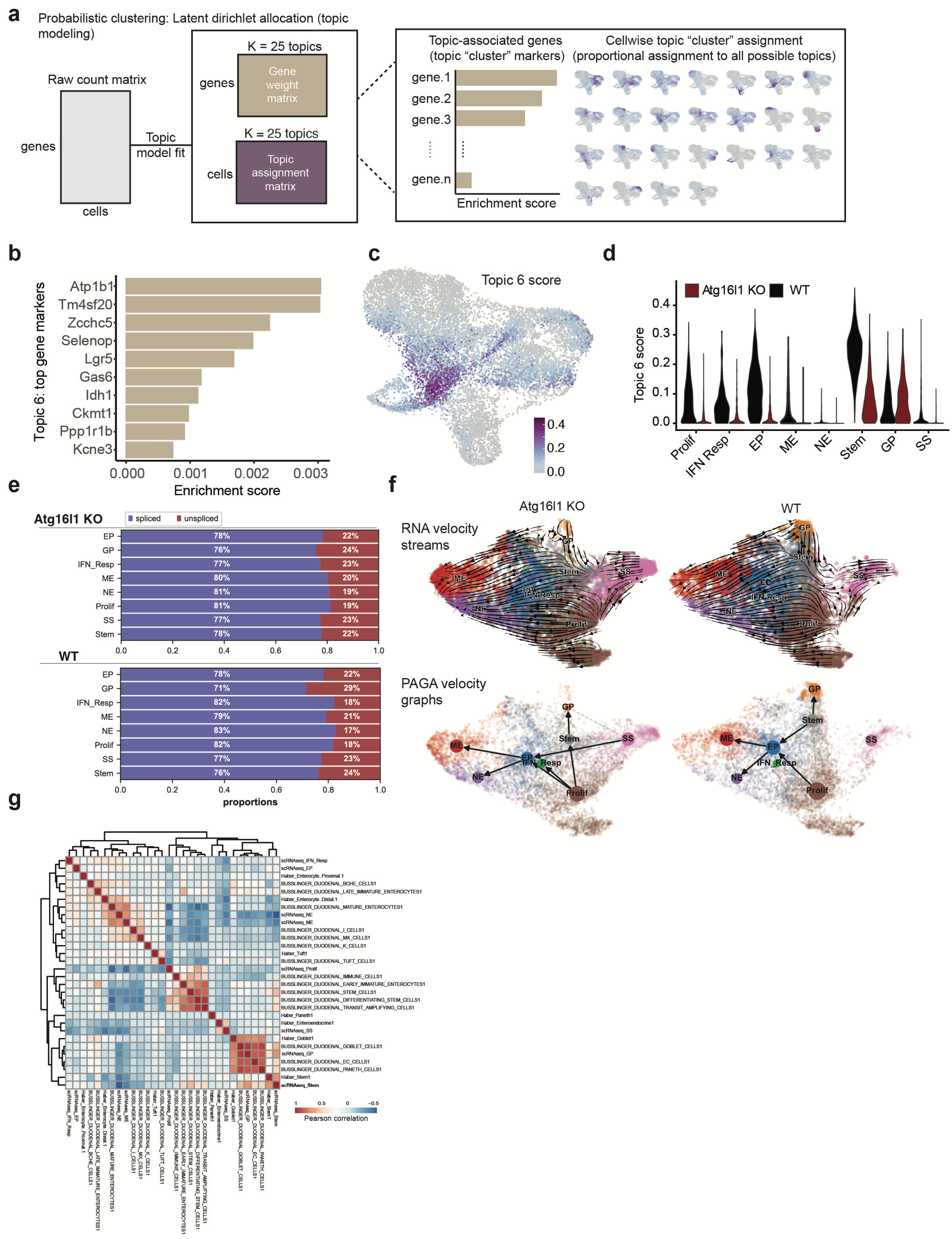

Supplementary Fig. 9: Loss of Atg16l1 alters the phenotype and composition of metastatic CRC cells in the liver.

a, Overall scheme for topic modeling analysis with organoid tumor scRNA-seq data. **b**, Top 10 genes enriched in topic 6 (the stemness topic). **c**, Topic 6 score visualized for individual CRC cells in UMAP dimensions. **d**, Violin plots showing topic 6 scores for CRC single cell clusters across WT and Atg16l1 KO conditions. **e**, Proportion of spliced and unspliced RNA-seq counts for each CRC single cell cluster. **f**, RNA velocity analysis for CRC single cells using scVelo: RNA velocity streams (top), and PAGA velocity graphs showing inferred trajectories (bottom). **g**, Heatmap of Pearson correlation coefficients comparing scores (FindModuleScore in Seurat) for the top 100 markers of each organoid scRNA-seq cluster with signatures derived from healthy human duodenum[3] and healthy murine small intestine[4].

Supplementary Figure 10

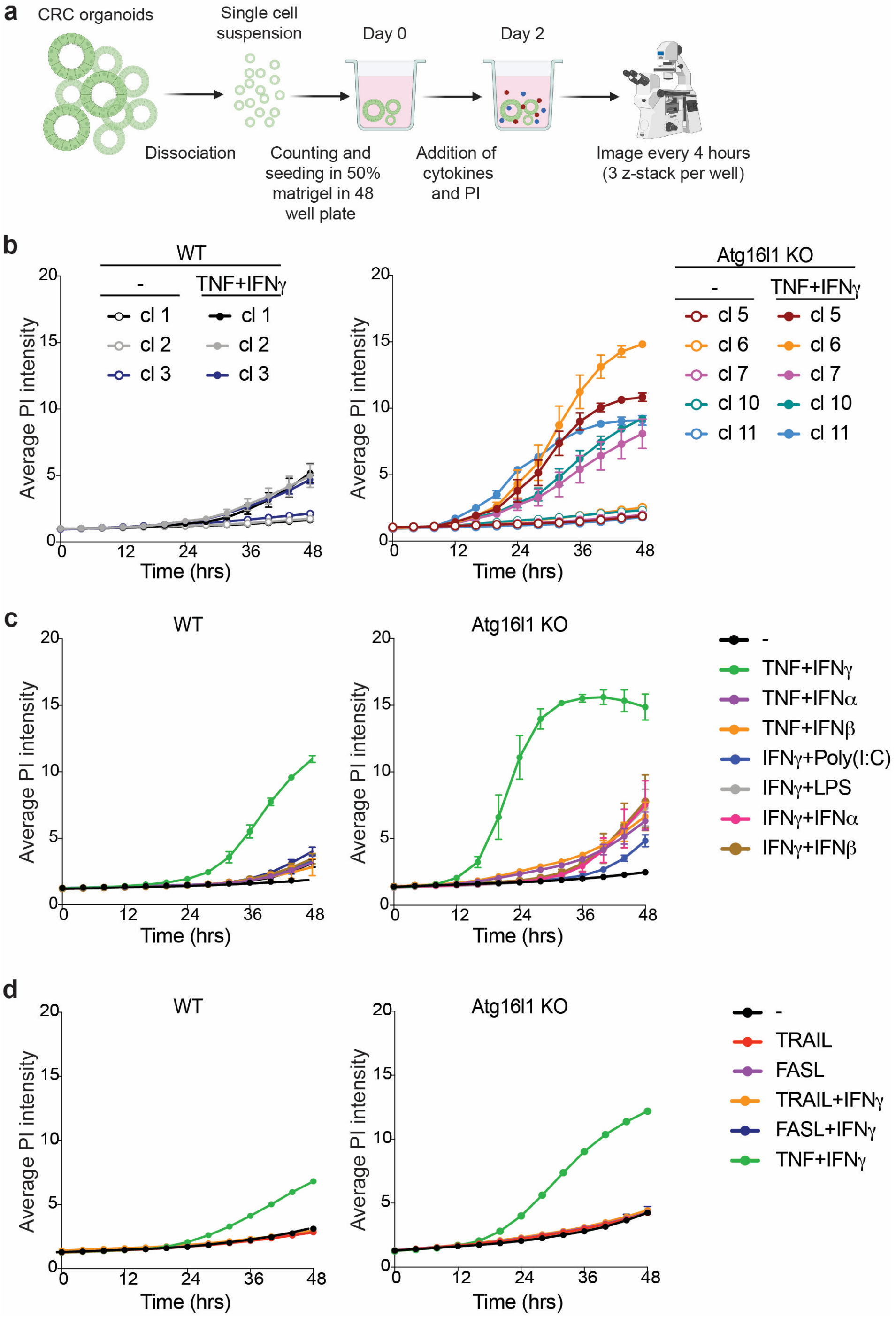

Supplementary Fig. 10: Cell death analysis of CRC organoids.
a, Schematic of experimental design. **b**, **c**, **d**, Cell death assayed by live-cell imaging of WT and Atg16l1 KO CRC organoids treated with combinations of TNF, IFNγ, IFNα, IFNβ, Poly(I:C), LPS, TRAIL, or FASL for 48 hours. Propidium Iodide (PI) staining is measured by fluorescence intensity/μm^2. Data represent 2–3 independent experiments with n=2–4 technical replicates per condition (see source data provided as a Source Data file).

Supplementary Figure 11

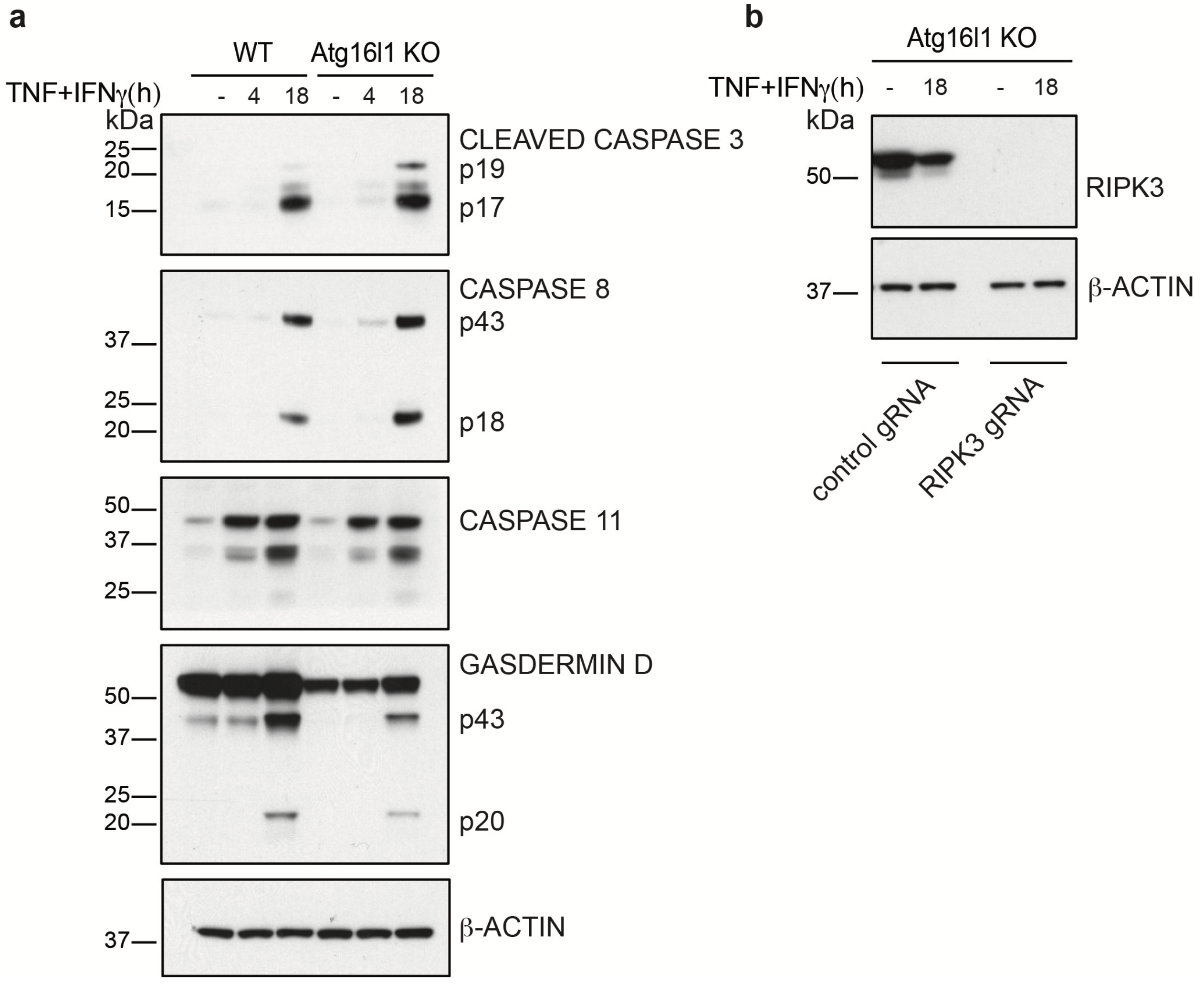

Supplementary Fig. 11: Analysis of cell death pathway components in WT and Atg16l1 KO CRC organoids.

a, **b**, Immunoblot analysis of the indicated proteins in WT, Atg16l1 KO and Atg16l1 KO Ripk3 KO CRC organoids stimulated with TNF and IFNγ for 4 or 18 hours. Data are representative of 2–3 independent experiments and 3 independent clones per genotype.

Supplementary Figure 12

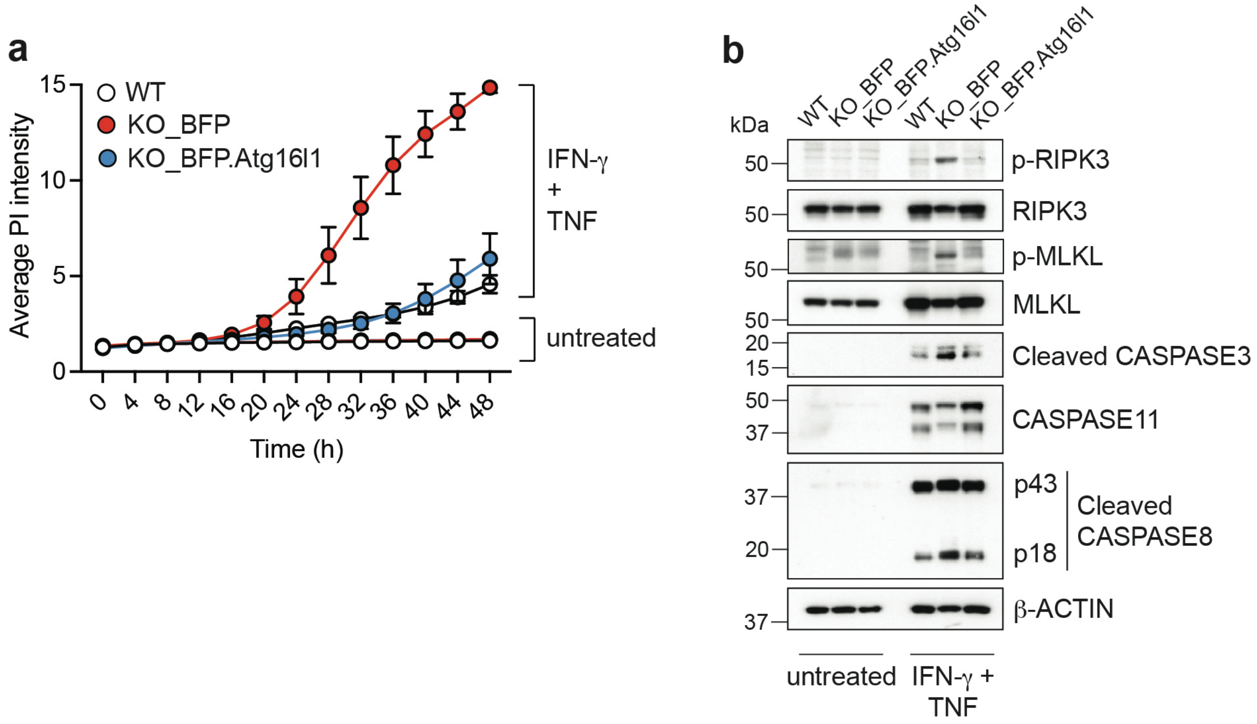

Supplementary Fig. 12: Analysis of cell death and cell death pathway components in Atg16l1 KO CRC organoids following re-expression of Atg16l1.

a, WT CRC organoids, Atg16l1 KO organoids expressing a control vector encoding BFP alone (KO_BFP), or Atg16l1 KO organoids expressing a vector encoding BFP and Atg16l1 (KO_BFP.Atg16l1), were analyzed in a cell death assay by live-cell imaging. Organoids were treated with TNF + IFNγ for 48 hours. PI staining was measured by fluorescence intensity/μm^2. **b**, Immunoblot analysis of the indicated phosphorylated and total proteins in WT, KO_BFP, or KO_BFP.Atg16l1 CRC organoids stimulated with TNF + IFNγ for 18 hours. Data are representative of two independent experiments.

Supplementary Figure 13

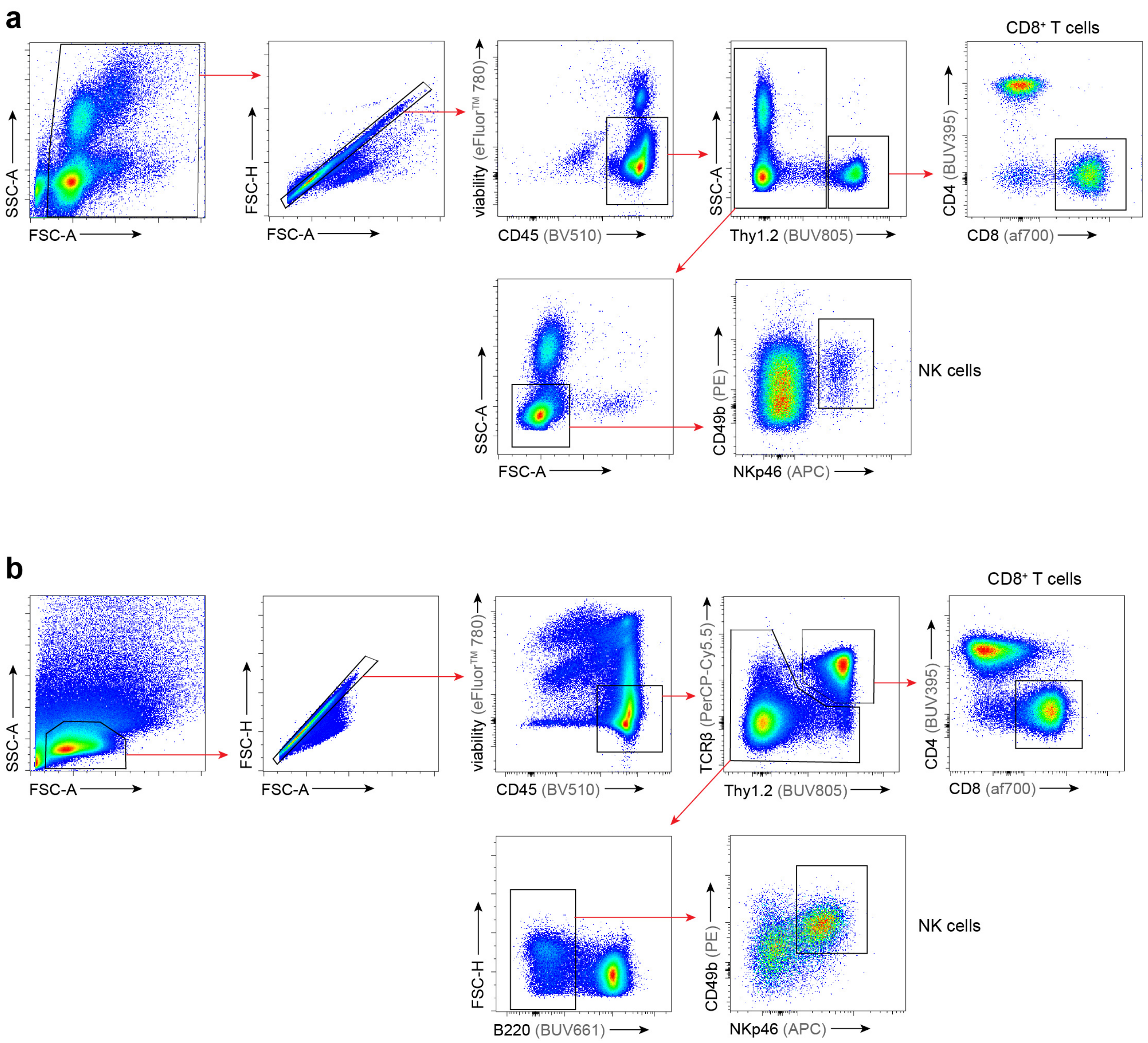

Supplementary Fig. 13. Flow cytometry gating strategy for detection of CD8⁺ T cell and NK cells.
To evaluate depletion of CD8⁺ T cells and NK cells (see Fig. 2f and Supplementary Fig. 8a-c), these cell types were identified using the indicated gating strategies for peripheral blood samples (**a**) and spleen samples (**b**).

Supplementary Table 1*: Median cutoff logrank test hazard ratios and (unadjusted) p-values for overall survival association of ATG genes in IMblaze370 KRAS mutant and wild type subcohorts

	**KRAS mutant**					
	**Atezolizumab monotherapy**		**Atezolizumab + Cobimetinib**		**Regorafenib**	
**GENE**	**P-value**	**Hazard ratio (95% CI)**	**P-value**	**Hazard ratio (95% CI)**	**P-value**	**Hazard ratio (95% CI)**
**ATG16L1**	***0.011 (risk)***	***2.51 (1.21-5.21)***	***0.0048 (risk)***	***1.94 (1.22-3.11)***	0.94	1.03 (0.512-2.06)
**ATG7**	0.45	0.769 (0.388-1.52)	0.51	0.856 (0.54-1.36)	0.79	1.1 (0.549-2.2)
**ATG10**	0.35	1.39 (0.692-2.77)	0.059	0.641 (0.402-1.02)	0.9	0.955 (0.462-1.97)
**ATG5**	0.23	1.51 (0.762-3)	0.12	1.45 (0.909-2.33)	0.89	1.05 (0.521-2.11)
**ATG12**	0.077	1.87 (0.925-3.78)	0.63	1.12 (0.705-1.77)	0.91	1.04 (0.511-2.12)
**ATG3**	0.64	1.18 (0.584-2.37)	0.54	1.16 (0.724-1.86)	0.26	1.51 (0.738-3.07)
**ATG4A**	0.81	0.919 (0.464-1.82)	0.37	1.23 (0.776-1.96)	0.56	0.809 (0.397-1.65)
**ATG4B**	0.096	1.79 (0.894-3.6)	0.38	1.23 (0.776-1.96)	0.66	1.18 (0.575-2.4)
**ATG4C**	0.7	0.867 (0.426-1.77)	0.29	0.782 (0.493-1.24)	0.83	0.924 (0.454-1.88)
**ATG4D**	***0.029 (risk)***	***2.26 (1.07-4.79)***	0.74	1.08 (0.682-1.71)	0.079	1.88 (0.919-3.87)

*Table continues on next page

Supplementary Table 1 ***continued***

	KRAS wild type					
	Atezolizumab monotherapy		Atezolizumab + Cobimetinib		Regorafenib	
GENE	P-value	Hazard ratio (95% CI)	P-value	Hazard ratio (95% CI)	P-value	Hazard ratio (95% CI)
ATG16L1	0.93	1.04 (0.461-2.33)	0.77	1.09 (0.596-2.01)	0.16	2.04 (0.735-5.67)
ATG7	0.36	0.684 (0.301-1.55)	0.85	0.943 (0.512-1.74)	0.58	0.749 (0.271-2.07)
ATG10	0.73	1.15 (0.521-2.53)	0.36	1.33 (0.726-2.43)	0.094	0.409 (0.139-1.2)
ATG5	0.068	0.459 (0.195-1.08)	0.23	1.45 (0.787-2.66)	0.55	0.737 (0.267-2.04)
ATG12	0.31	1.58 (0.647-3.84)	0.39	1.3 (0.709-2.39)	0.95	1.04 (0.375-2.86)
ATG3	0.22	0.592 (0.256-1.37)	***0.011 (risk)***	***2.21 (1.19-4.11)***	0.57	0.736 (0.251-2.16)
ATG4A	0.32	1.5 (0.667-3.37)	0.5	1.23 (0.672-2.25)	0.85	1.11 (0.391-3.14)
ATG4B	0.62	1.24 (0.533-2.89)	0.49	0.81 (0.443-1.48)	***0.0042 (risk)***	***4.74 (1.48-15.2)***
ATG4C	0.85	0.923 (0.402-2.12)	***0.0084 (risk)***	***2.3 (1.22-4.33)***	0.19	0.507 (0.179-1.43)
ATG4D	0.2	1.75 (0.734-4.16)	0.43	1.28 (0.698-2.33)	0.64	1.29 (0.439-3.77)

Supplementary Table 2: Association of core autophagy genes ATG7, ATG10, ATG5, ATG12, ATG4A, ATG4B, ATG4C, ATG4D with patient outcome in late-stage CRC across observational studies.

**Dataset**	**OS (n)**	**DSS (n)**	**DFS(n)**	**MSS/MSI (n)**	**Non-MSI-high KRASmt (n)**	**Prognostic association with ATG genes?**
GSE17536 stage IV	39	39	10	unknown	unknown	**DSS:** ATG16L1 associated significantly with poor DSS (P<0.05); no association for other ATG genes<br><br>**OS:** ATG16L1 associated significantly with poor OS (P<0.05); no association for other ATG genes
GSE39582 stage IV	61	NA	61	56 MSS, 2 MSI, 3 unknown	27	**DFS:** ATG7, ATG10, ATG4A associated significantly with poor DFS (P<0.05); ATG4B/C/D, ATG5, ATG12, ATG16L1 not associated with DFS<br><br>**OS:** No significant associations
TCGA stage IV	86	86	NA	0 MSI-high, 13 MSI-low, 27 MSS, 46 unknown	33	**DSS:** No significant associations<br>**OS:** No significant associations

OS, overall survival; DSS, disease-specific survival; DFS, disease-free survival. Median expression cutoff was used for survival analysis.

References

1. Melia, T. J., Lystad, A. H. & Simonsen, A. Autophagosome biogenesis: From membrane growth to closure. *J. Cell Biol.* **219**, e202002085 (2020).

2. Lim, J. & Murthy, A. Targeting Autophagy to Treat Cancer: Challenges and Opportunities. *Front. Pharmacol.* **11**, 590344 (2020).

3. Busslinger, G. A. *et al.* Human gastrointestinal epithelia of the esophagus, stomach, and duodenum resolved at single-cell resolution. *Cell Rep.* **34**, 108819 (2021).

4. Haber, A. L. *et al.* A single-cell survey of the small intestinal epithelium. *Nature* **551**, 333–339 (2017).
