# Supplementary material for: Tumor-intrinsic expression of the autophagy gene Atg16l1 suppresses anti-tumor immunity in colorectal cancer
Source: Nat Commun. 2023 Sep 23;14:5945. doi: 10.1038/s41467-023-41618-7 (PMC10517947; doi:10.1038/s41467-023-41618-7)
Supplement: Supplementary file 3 — Reporting Summary [file 41467_2023_41618_MOESM3_ESM.pdf]

nature portfolio

Corresponding author(s): Aditya Murthy, Nathaniel R. West

Last updated by author(s): Aug 31, 2023

Reporting Summary

Nature Portfolio wishes to improve the reproducibility of the work that we publish. This form provides structure for consistency and transparency in reporting. For further information on Nature Portfolio policies, see our Editorial Policies and the Editorial Policy Checklist.

Statistics

For all statistical analyses, confirm that the following items are present in the figure legend, table legend, main text, or Methods section.

n/a	Confirmed	
☐	☒	The exact sample size (*n*) for each experimental group/condition, given as a discrete number and unit of measurement
☐	☒	A statement on whether measurements were taken from distinct samples or whether the same sample was measured repeatedly
☐	☒	The statistical test(s) used AND whether they are one- or two-sided<br>*Only common tests should be described solely by name; describe more complex techniques in the Methods section.*
☐	☒	A description of all covariates tested
☐	☒	A description of any assumptions or corrections, such as tests of normality and adjustment for multiple comparisons
☐	☒	A full description of the statistical parameters including central tendency (e.g. means) or other basic estimates (e.g. regression coefficient) AND variation (e.g. standard deviation) or associated estimates of uncertainty (e.g. confidence intervals)
☐	☒	For null hypothesis testing, the test statistic (e.g. *F*, *t*, *r*) with confidence intervals, effect sizes, degrees of freedom and *P* value noted<br>*Give P values as exact values whenever suitable.*
☒	☐	For Bayesian analysis, information on the choice of priors and Markov chain Monte Carlo settings
☒	☐	For hierarchical and complex designs, identification of the appropriate level for tests and full reporting of outcomes
☐	☒	Estimates of effect sizes (e.g. Cohen's *d*, Pearson's *r*), indicating how they were calculated

Our web collection on statistics for biologists contains articles on many of the points above.

Software and code

Policy information about availability of computer code

Data collection	Flow cytometry data were collected using BD FACSDiva software. Organoid death was measured using the Incucyte® SX5 Live-Cell Analysis System (Essen Bioscience) and was imaged and analyzed using the Organoid Culture QC module.
Data analysis	Data analysis was performed with Rstudio (R 4.1.0, Bioconductor 3.13) and GraphPad Prism 8 except when noted otherwise. Python 3.8.6 was used for RNA velocity analysis. Matlab r2019a was used for H&E image analysis and cell death measurements as specified in Methods.

For manuscripts utilizing custom algorithms or software that are central to the research but not yet described in published literature, software must be made available to editors and reviewers. We strongly encourage code deposition in a community repository (e.g. GitHub). See the Nature Portfolio guidelines for submitting code & software for further information.

Data

Policy information about availability of data

All manuscripts must include a data availability statement. This statement should provide the following information, where applicable:

- Accession codes, unique identifiers, or web links for publicly available datasets
- A description of any restrictions on data availability
- For clinical datasets or third party data, please ensure that the statement adheres to our policy

Raw and processed data from in vitro organoid bulk RNA sequencing, and in vivo organoid single cell RNA sequencing have been submitted to Gene Expression Omnibus with accession number GSE192515. Raw data from in vitro organoid whole exome sequencing have been deposited to Sequence Read Archive with accession number PRJNA790973.

IMblaze370 bulk RNA sequencing raw data have been submitted to the European Genome-Phenome Archive (EGAS00001005952). Requests for the exploratory

biomarker data underlying this publication requires a detailed, hypothesis-driven statistical analysis plan that is collaboratively developed by the requestor and company subject matter experts. Direct such requests to Y.Y. (yan.yibing@gene.com) for consideration.

Further details on Roche's Global Policy on the Sharing of Clinical Information and how to request access to related clinical study documents are available online (https://go.roche.com/data_sharing). Anonymised records for individual patients across more than one data source external to Roche can not, and should not, be linked due to a potential increase in risk of patient re-identification.

Additional publicly available datasets used in this study (accessible via Gene Expression Omnibus) include GSE146771, GSE17536, and GSE39582. Bulk tumor RNAseq data from TCGA are available from TCGA website (https://www.cancer.gov/ccg/research/genome-sequencing/tcga).

Field-specific reporting

Please select the one below that is the best fit for your research. If you are not sure, read the appropriate sections before making your selection.

- [x] Life sciences
- [ ] Behavioural & social sciences
- [ ] Ecological, evolutionary & environmental sciences

For a reference copy of the document with all sections, see nature.com/documents/nr-reporting-summary-flat.pdf

Life sciences study design

All studies must disclose on these points even when the disclosure is negative.

Sample size	For pre-clinical and in vitro studies, no sample size calculation was performed. Sample sizes for individual experiments were chosen on the basis of prior experience with specific model systems (e.g. n=10 mice per group in syngeneic tumor growth studies is sufficient to generate statistically meaningful data, given the known mouse-to-mouse variability in tumor control). For IMBlaze370, all samples which passed RNA sequencing quality controls detailed in Methods were used for analysis.
Data exclusions	Gene expression datasets: All samples which passed RNA sequencing quality controls were used. No sample exclusion was performed for in vivo or in vitro phenotypic studies.
Replication	Replication was verified in individual biological replicates and studies as detailed in each figure legend.
Randomization	No sample randomization was performed for pre-clinical studies, as genetically modified tumor organoids were used in pre-determined experimental groups.<br>IMBlaze370 study design is detailed in Eng, C. et al. Atezolizumab with or without cobimetinib versus regorafenib in previously treated metastatic colorectal cancer (IMblaze370): a multicentre, open-label, phase 3, randomised, controlled trial. Lancet Oncol 20, 849–861 (2019).<br>As described in the original manuscript: "We used permuted-block randomisation (block size four) to assign patients (2:1:1) via an interactive voice and web response system to atezolizumab (840 mg intravenously every 2 weeks) plus cobimetinib (60 mg orally once daily for days 1–21 of a 28-day cycle), atezolizumab monotherapy (1200 mg intravenously every 3 weeks), or regorafenib (160 mg orally once daily for days 1–21 of a 28-day cycle)."
Blinding	No blinding of experimental groups was performed, as different genotypes (e.t., Wt vs. Atg16l1-KO) were directly compared for phenotypic differences.

Reporting for specific materials, systems and methods

We require information from authors about some types of materials, experimental systems and methods used in many studies. Here, indicate whether each material, system or method listed is relevant to your study. If you are not sure if a list item applies to your research, read the appropriate section before selecting a response.

Materials & experimental systems

n/a	Involved in the study	
☐	☒	Antibodies
☐	☒	Eukaryotic cell lines
☒	☐	Palaeontology and archaeology
☐	☒	Animals and other organisms
☒	☐	Human research participants
☐	☒	Clinical data
☒	☐	Dual use research of concern

Methods

n/a	Involved in the study	
☒	☐	ChIP-seq
☐	☒	Flow cytometry
☒	☐	MRI-based neuroimaging

Antibodies

Antibodies used	ATG16L1: 1/4000; clone 1F12; MBL international Cat# M150-3; RRID AB_1278758<br>MLKL: 1/1000; Genentech, Inc; clone 1G12<br>p-MLKL (pSer345): 1/1000; clone D6E3G; Cell Signaling Technology Cat# 37333

	RIPK3: 1/2000; Novus Biologicals Cat#: NBP1-77299<br>pRIPK3 (pThr231/Ser232): 1/1000; Genentech, Inc; clone GEN-135-35-9<br>GSDMD: 1/4000; Genentech, Inc; clone GN20-13<br>CALCOCO1: 1/4000; Proteintech Cat# 19843–1-AP<br>Sqstm1/p62: 1/4000; Cell Signaling Technology Cat# 5114<br>LC3A/B: 1/4000; clone D3U4C; Cell Signaling Technology Cat# 12741<br>TAX1BP1: 1/4000; clone EPR13287(B); Abcam Cat # ab176572<br>Cleaved Caspase-3: 1/2000; clone 5A1E; Cell Signaling Technology Cat# 9664<br>Cleaved caspase-8: 1/1000; clone D35G2; Cell Signaling Technology Cat# 9429<br>Caspase 11: 1/1000; clone 17D9; Cell Signaling Technology Cat# 14340<br>beta-ACTIN: 1/10000; clone D6A8; Cell Signaling Technology Cat# 3700<br>Rabbit IgG-HRP: 1/4000; Cell Signaling Technology Cat# 7074<br>Mouse IgG-HRP: 1/4000; Cell Signaling Technology Cat# 7076<br>Rat Ig-HRP: 1/4000; Cell Signaling Technology Cat# 7077<br>CD8 IgG2b depleting antibody: clone 2.43; produced at Genentech<br>NK1.1 IgG2a depleting antibody: clone PK136; Bio X Cell Cat# BE0036<br>Mouse IgG2a: produced at Genentech<br>Mouse IgG2b: produced at Genentech<br>CD16/CD32 Fc block: 5 ug/ml; clone 2.4G2; BD Biosciences Cat# 553141<br>CD45-BV510: 2 ug/ml; clone 30-F11; Biolegend Cat# 103138<br>TCRb-PerCP-Cy5.5: 2 ug/ml; clone H57-597; Biolegend Cat# 109228<br>CD4-BUV395: 2 ug/ml; clone GK1.5; BD Biosciences Cat# 563790<br>CD8-af700: 5 ug/ml; clone KT15; Bio-Rad Cat# MCA609A700<br>Nkp46-APC: 1 ug/ml; clone 29A1.4; Biolegend Cat# 137607<br>CD49b-PE: 2 ug/ml; clone DX5; Biolegend Cat# 108908<br>B220-BUV661: 2 ug/gml; clone RA3-6B2; BD Biosciences Cat# 612972<br>Thy1.2-BUV805: 1 ug/ml; clone 53-2.1; BD Biosciences Cat# 741908
Validation	Data provided in the manuscript:<br>ATG16L1, RIPK3: western blot of CRISPR-KO cells.<br>CALCOCO1, Sqstm1/p62, LC3A/B, TAX1BP1: Western blot of ATG16L1 ko CRISPR-KO cells.<br>p-RIPK3, p-MLKL, cleaved caspase 3, cleaved caspase 8, caspase 11, Gasdermin D: Western blot of organoids under programmed cell death conditions.<br><br>CD45, TCRb, CD4, NKp46, CD49b, B220, Thy1.2 fluorescently conjugated antibodies: Flow cytometry assessment of murine PBMC and splenocytes.<br>CD8 and NK1.1 depleting antibodies also validated by detection of CD8 T cells and NK cells with non-competing antibodies (by flow cytometry) following antibody-mediated cell depletion.<br><br>Data provided in prior publication (Lim et al, eLife, 2019, https://doi.org/10.7554/eLife.44452):<br>ATG16L1: western blot of cells from KO mice.<br>RIPK3, MLKL, GSDMD, TAX1BP1, CALCOCO1, Sqstm1/p62: western blot of CRISPR-KO cells.<br>pRIPK3: western blot of macrophages under cell death conditions, and reversal of staining with necroptosis inhibitor Nec1.<br><br>All listed antibodies (not produced by Genentech) also have validation provided in manufacturer websites.

Eukaryotic cell lines

Policy information about cell lines

Cell line source(s)	Colorectal cancer organoids were generated for this study via CRISPR-Cas9 engineering as detailed in Methods.
Authentication	Authentication for relevant genetic modifications were performed via whole-exome sequencing (APC loss, KRAS G12D, SMAD4 loss, Tp53 loss; transformed cells termed 'AKPS'). Knockout of relevant genes were confirmed by immunoblotting.
Mycoplasma contamination	Parental AKPS organoid cells were tested for Mycoplasma contamination and confirmed to be negative.
Commonly misidentified lines<br>(See ICLAC register)	N/A - no highly transformed cell lines were used in the study.

Animals and other organisms

Policy information about studies involving animals; ARRIVE guidelines recommended for reporting animal research

Laboratory animals	Strains used in this manuscript:<br>B57BL/6J: Jackson lab cat#: 000664<br>NOD.Cg-PrkdcscidIl2rgtm1Wjl/SzJ: Jackson lab colony 005557<br>B6.129S7-IFNgtm1TS/J: Jackson lab cat #:002287<br><br>Female mice (age 6-12 weeks) were used for this study.

Wild animals	No wild animals were used in this study
Field-collected samples	No field-collected samples were used in this study
Ethics oversight	Animal studies were approved by Genentech's Institutional Animal Care and Use Committee and adhere to the NRC Guidelines for the Care and Use of Laboratory Animals.

Note that full information on the approval of the study protocol must also be provided in the manuscript.

Clinical data

Policy information about clinical studies
All manuscripts should comply with the ICMJE guidelines for publication of clinical research and a completed CONSORT checklist must be included with all submissions.

Clinical trial registration	NCT02788279
Study protocol	Previously published in: Eng, C. et al. Atezolizumab with or without cobimetinib versus regorafenib in previously treated metastatic colorectal cancer (IMblaze370): a multicentre, open-label, phase 3, randomised, controlled trial. Lancet Oncol 20, 849–861 (2019).
Data collection	Previously published in: Eng, C. et al. Atezolizumab with or without cobimetinib versus regorafenib in previously treated metastatic colorectal cancer (IMblaze370): a multicentre, open-label, phase 3, randomised, controlled trial. Lancet Oncol 20, 849–861 (2019).<br><br>Bulk RNA-Seq analysis is described in Methods of the currently submitted manuscript.
Outcomes	Previously published in: Eng, C. et al. Atezolizumab with or without cobimetinib versus regorafenib in previously treated metastatic colorectal cancer (IMblaze370): a multicentre, open-label, phase 3, randomised, controlled trial. Lancet Oncol 20, 849–861 (2019).

Flow Cytometry

Plots

Confirm that:

☒ The axis labels state the marker and fluorochrome used (e.g. CD4-FITC).

☒ The axis scales are clearly visible. Include numbers along axes only for bottom left plot of group (a 'group' is an analysis of identical markers).

☒ All plots are contour plots with outliers or pseudocolor plots.

☒ A numerical value for number of cells or percentage (with statistics) is provided.

Methodology

Sample preparation	See Methods: Tissue processing
Instrument	See Methods: Flow cytometry
Software	See Methods: Flow cytometry
Cell population abundance	Provided in Extended Data Fig. 8
Gating strategy	Provided in Extended Data Fig. 8

☒ Tick this box to confirm that a figure exemplifying the gating strategy is provided in the Supplementary Information.
